# Poly‐d,l‐lactic acid‐enhanced atrophic scar treatment via transdermal microjet drug delivery in Asians

**DOI:** 10.1111/srt.13762

**Published:** 2024-06-20

**Authors:** Suk Bae Seo, Jovian Wan, Lisa Kwin Wah Chan, Kar Wai Alvin Lee, Soo‐Bin Kim, Kyu‐Ho Yi

**Affiliations:** ^1^ SeoAhSong Dermatologic Clinic Seoul South Korea; ^2^ Asia‐Pacific Aesthetic Academy Hong Kong Hong Kong; ^3^ EverKeen Medical Centre Hong Kong Hong Kong; ^4^ Department of Oral Biology Human Identification Research Institute Division in Anatomy and Developmental Biology BK21 FOUR Project Yonsei University College of Dentistry Seoul South Korea; ^5^ Maylin Clinic (Apgujeong) Seoul South Korea

**Keywords:** acne scar, acne vulgaris, l‐lactic acid, needle‐free injector, poly‐d, scar

## Abstract

**Background:**

Acne vulgaris often results in permanent scars, with atrophic scars being the most common type and posing a significant therapeutic challenge due to their prevalence and impact on patients' quality of life. Various treatment options exist, including the use of poly‐d,l‐lactic acid delivered via different methods.

**Objective:**

This study aimed to assess the efficacy and safety of poly‐d,l‐lactic acid delivered via laser‐assisted needle‐free microjet injection for treating atrophic scars.

**Methods:**

Five Korean participants with atrophic facial scars were recruited. Poly‐d,l‐lactic acid solution was administered via the Mirajet system in five sessions, with clinical assessments conducted at baseline, before each session, and at 12‐week and 22‐week follow‐ups. Outcome measures included the Global Aesthetic Improvement Scale and patient satisfaction scores.

**Results:**

Positive results were observed at the 12‐week and 22‐week follow‐ups, with high patient satisfaction and improvements in atrophic scars and skin texture. Mild discomfort and transient side effects were reported, with no adverse events observed during the follow‐up period.

**Conclusion:**

Poly‐d,l‐lactic acid delivered by a laser‐assisted needle‐free microjet injector was judged to be effective for improving atrophic the facial area. Further research, particularly through randomized controlled trials, is needed to validate these findings and assess the longer‐term safety and sustainability of outcomes.

## 1 INTRODUCTION

Inflammatory acne vulgaris lesions often lead to permanent scars, which can be categorised as atrophic, hypertrophic, or keloidal.^1^ Atrophic scars, the most prevalent type, present a significant therapeutic challenge due to their high prevalence and impact on patients' quality of life.^2^, ^3^ They develop due to irregular collagen production and degradation during the healing process, leading to an enzymatic breakdown of collagen fibres in the dermis.^4^ Atrophic scars are commonly classified as ice pick, rolling, or boxcar scars.^2^ Treatment options vary depending on scar type and the available therapies, including laser therapy, chemical peels, punch excision, subcision, dermabrasion, thread lifting, fat transplantation, micro‐needling, injectable fillers and combinations thereof.^3^, ^5^, ^6^

Poly‐d,l‐lactic acid (PDLLA) has been studied as an injectable filler for addressing acne scars through various methods such as microneedle fractional radiofrequency, manual injections, and needle‐free jet injections.^6^, ^7^, ^8^, ^9^ Effective transdermal drug delivery methods are crucial in dermatological procedures. The Mirajet device, employing laser energy, has emerged as a significant breakthrough, delivering drugs quickly and evenly without causing substantial damage.^10^ This technology employs high‐speed jet pressure, creating vibrations and shocks that stimulate mechanotransduction within cells.^11^ The device achieves the highest speed among recent needleless injection systems. It focuses laser energy on a medium to generate a microjet, selecting appropriate laser parameters and a suitable medium. The drug is stored in a sub‐chamber separated by an elastic membrane, ejected at speeds sufficient to penetrate the skin.^12^, ^13^ The system ensures sustainability and reproducibility, with ongoing efforts to enhance its durability and efficiency. Clinically, it allows the delivery of various drugs with minimal discomfort and downtime, offering promising results, particularly with PDLLA preparations. In this study, we use the laser‐assisted needle‐free microjet, Mirajet system, for the delivery of PDLLA to target atrophic scars.

## 2 MATERIAL AND METHOD

### 2.1 Patients

Five Korean participants, aged between 33 and 52, with atrophic facial scars, were recruited for this study. One of the participants was male, while the remaining were female. All participants sought treatment for atrophic scars and presented with Fitzpatrick skin type III. The research was conducted in accordance with the principles outlined in the Declaration of Helsinki. This study obtained a Non‐Interventional Study waiver from the local Ethics Committee to proceed with data collection.

### 2.2 Inclusion and exclusion criteria

Inclusion criteria consisted individuals with facial atrophic scars, while exclusion criteria included individuals under 18 years of age, individuals with active acne vulgaris, pregnant or lactating women, women taking contraceptive pills within the study period or in the past year, individuals currently undergoing atrophic scar therapy, those who had received facial aesthetic treatments including energy‐based devices within the past 6 months, and individuals with a history of hypersensitivity of allergic reactions to PDLLA.

### 2.3 Poly‐d,l‐lactic acid preparation

A PDLLA 50 mg/hyaluronic acid (HA) 7.5 mg filler was dissolved in 10 mL of normal saline and vortexing for 2 h before use.

### 2.4 Treatment protocol

The treatment regimen involved administration of PDLLA solution (Juvelook, VAIM Global, South Korea) administered via the microjet injection system (Mirajet, JSK Inc., South Korea). Patient underwent sessions twice a month for a total of five sessions, with the injections performed by a single dermatologist until a total of 3cc of diluted PDLLA (Juvelook, VAIM Inc., Seoul, Korea) was delivered. The injection technique employed a 200 𝜇m‐sized nozzle, operating at 30 Hz and delivering 14000–15000 pulses per session. Each pulse contained 0.2–0.3 𝜇L of the drug, comprising 16 mg of PDLLA in 3cc of normal saline. Each atrophic scar lesion received two to three consecutive injections. The end‐point of treatment was determined by the presence of papules with focal blanching and central pinpoint bleeding, alongside mild swelling at the injection sites.

To improve patient comfort, a topical anesthetic cream containing lidocaine and prilocaine was applied to the treatment area 30 min before the procedure. Additionally, a mixture of lidocaine and epinephrine was added to the solution to further reduce bleeding and discomfort, administered at low energy levels prior to the main treatment.

During the procedure, overlapping injections were employed instead of dropping the nozzle, which proved effective in reducing pain and facilitating micro‐incisions. Occasionally, minor bleeding occurred when administering strong energy with rounded particles like PDLLA, necessitating immediate pressure treatment to reduce the bruising duration. Effective pressure treatment post‐procedure can help minimise the duration of residual bruising. Minor bleeding was addressed by gently wiping the area with gauze soaked in a 10% dilution.

### 2.5 Clinical assessment

Clinical digital photographs and three‐dimensional (3D) images were taken at baseline, before each procedural session, and during follow‐up appointments, which were scheduled 12 weeks after the final treatment. Clinical photographs were taken with a digital camera and 3D images were acquired using a 3D camera (LifeViz Infinity; QuantifiCare, Biot, France). All images were captured under consistent positioning and lighting conditions in the photography room.

Patient improvement was evaluated 12 weeks and 22 weeks after the last treatment using the Global Aesthetic Improvement Scale (GAIS) by two independent physicians who were blinded to the study details. Scores ranging from 1 to 5 were assigned. Patient satisfaction with aesthetic outcomes was self‐assessed using a 4‐point scale of 0 to 3 at the same 12‐week and 22‐week follow‐up, with higher scores indicating higher levels of satisfaction.

## 3 RESULTS

**FIGURE 1:**
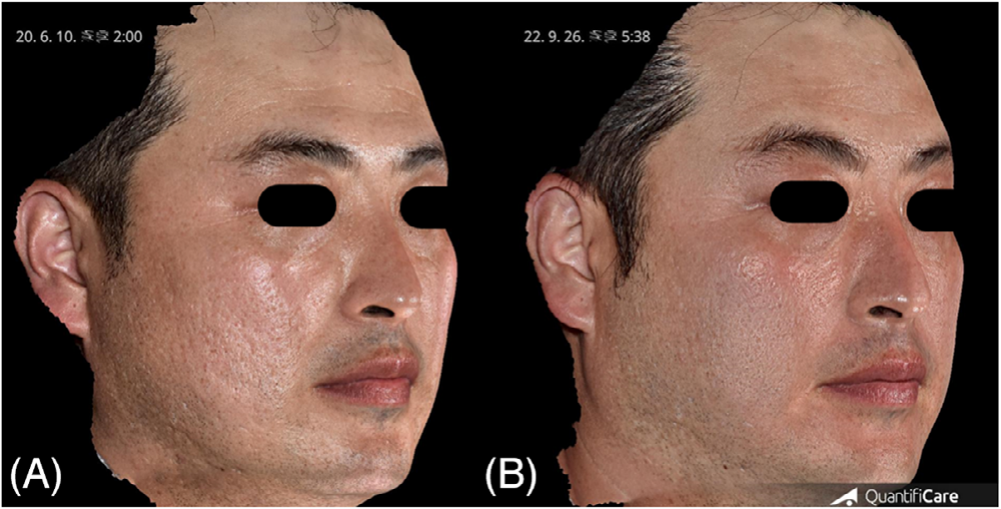
Three‐dimensional images of case 1 (male, 36 years), demonstrating marked improvement in facial atrophic scars following treatment of poly‐d,l‐lactic acid via laser‐assisted needle‐free microjet. The images depict the condition before (A) and 22 weeks after the final treatment (B). Three‐dimensional images were acquired using a three‐dimensional camera (LifeViz Infinity; QuantifiCare, Biot, France).

**FIGURE 2:**
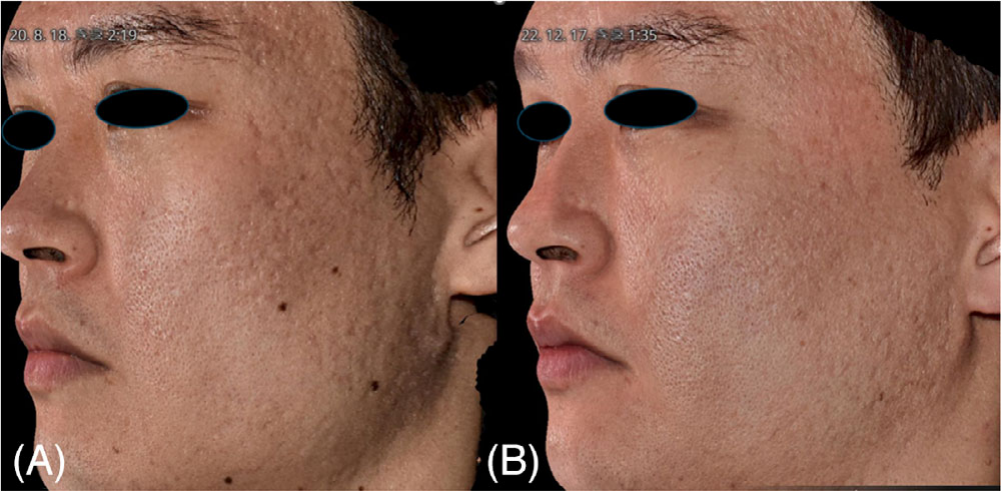
Three‐dimensional images of case 2 (female, 33 years), demonstrating improvement in facial atrophic scars following treatment of poly‐d,l‐lactic acid via laser‐assisted needle‐free microjet. The images depict the condition before (A) and 22 weeks after the final treatment (B). Three‐dimensional images were acquired using a three‐dimensional camera (LifeViz Infinity; QuantifiCare, Biot, France).

**FIGURE 3:**
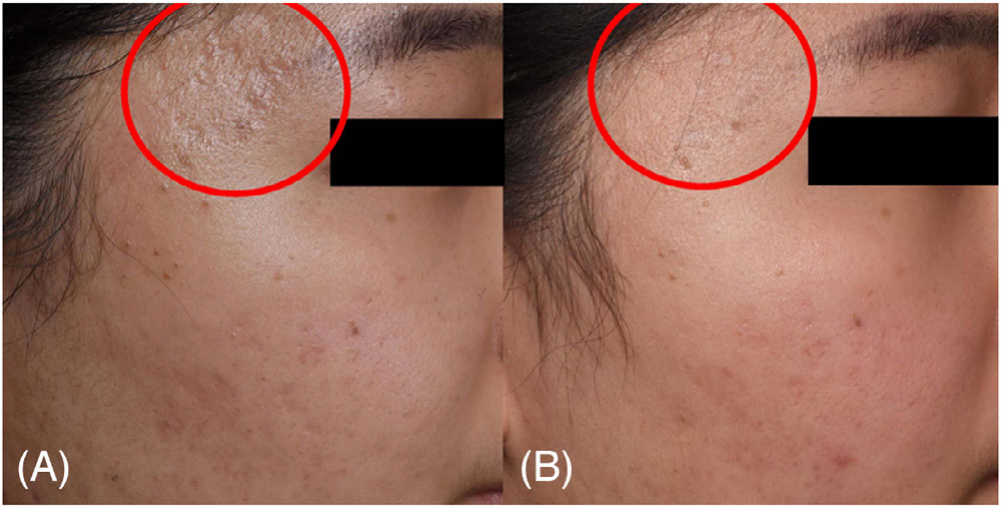
Close‐up three‐dimensional images of the right temple region of case 3 (female, 52 years), demonstrating improvement in facial atrophic scars following treatment of poly‐d,l‐lactic acid via laser‐assisted needle‐free microjet. The images depict the condition before (A) and 22 weeks after the final treatment (B). Three‐dimensional images were acquired using a three‐dimensional camera (LifeViz Infinity; QuantifiCare, Biot, France). The figure is taken by Hong‐Seok Kim.

**FIGURE 4:**
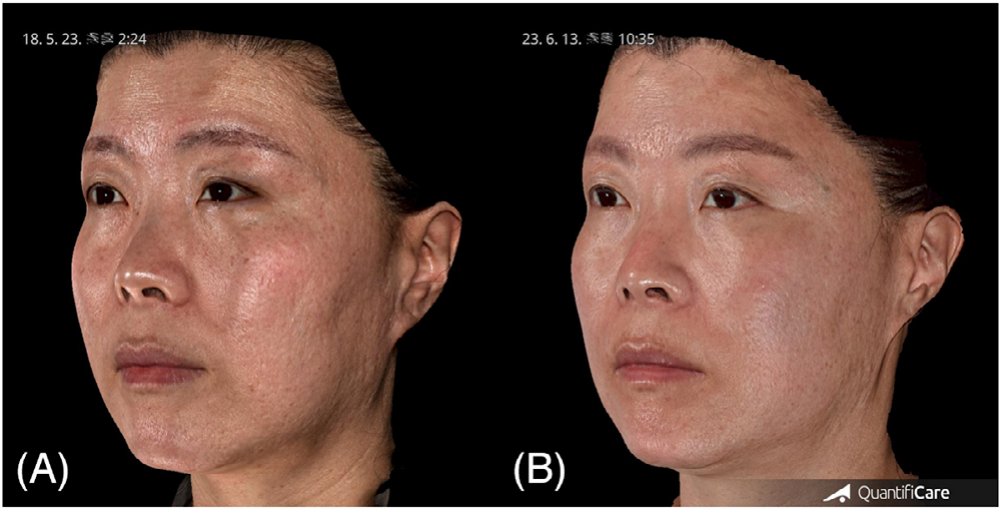
Three‐dimensional images of case, demonstrating facial atrophic scar treatment pre‐treatment (A) and 12‐week post‐final treatment (B).

**FIGURE 5:**
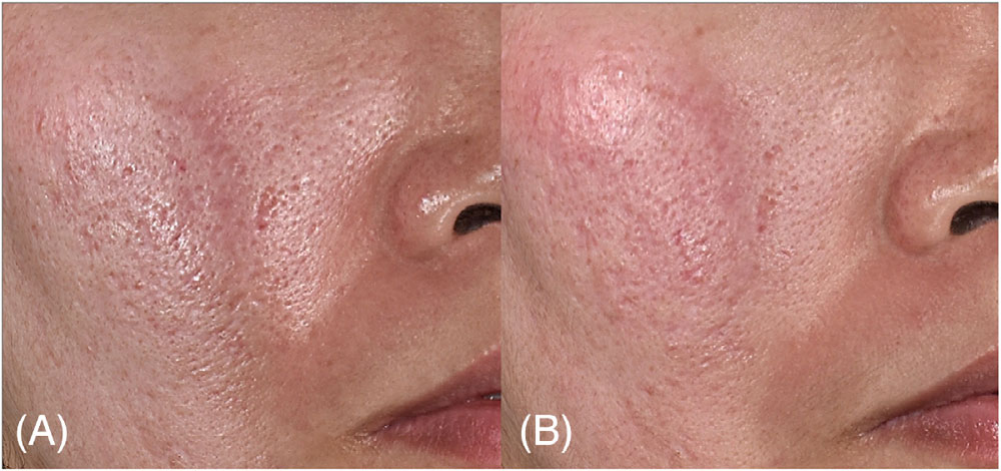
Close‐up three‐dimensional images of the right cheek of case 5 (female, 44 years), demonstrating marked improvement in facial atrophic scars following treatment of poly‐d,l‐lactic acid via laser‐assisted needle‐free microjet. The images depict the condition before (A) and 22 weeks after the final treatment (B). Three‐dimensional images were acquired using a three‐dimensional camera (LifeViz Infinity; QuantifiCare, Biot, France).

All assessments from both the GAIS and the patient satisfaction scale indicated positive results at the 12‐week follow‐up after the final treatment, with scores remaining stable at the 22‐week follow‐up. All patients expressed high satisfaction with the improvement of their atrophic scars and noted enhancements in skin texture as well. For a summary of the results, please refer to Table 1. All patients reported slight discomfort during the procedure, with pain scores ranging from 2 to 3 on a scale of 0 to 5. Following each treatment, patients experienced mild oedema and erythema, which persisted around 24 h. Mild petechiae were observed in all patients but resolved spontaneously within 72 h. No adverse events related to the product, such as nodule formation or local inflammation, were observed during the 22‐week follow‐up period (Figures 1, 2, 3, 4, and 5).

**TABLE 1:** A summary of the assessment scores used in the study. The Global Aesthetic Improvement Scale (GAIS) scores were evaluated independently by two blinded physicians 12 weeks after the final treatment, reflecting the overall improvement in aesthetic appearance. Patient satisfaction scores, obtained through self‐reporting using a 4‐point questionnaire, indicate the level of satisfaction with the treatment outcome.

Participant	GAIS assessment by physician 1 at 12 weeks post‐final treatment	GAIS assessment by physician 1 at 22 weeks post‐final treatment	GAIS assessment by physician 2 at 12 weeks post‐final treatment	GAIS assessment by physician 2 at 22 weeks post‐final treatment	Patient self‐evaluation score at 12 weeks post‐final treatment	patient self‐evaluation score at 22 weeks post‐final treatment
**1**	2	2	2	2	3	3
**2**	2	2	2	2	2	2
**3**	2	2	2	2	2	2
**4**	2	2	2	2	3	3
**5**	2	2	3	2	2	2

## 4 DISCUSSION

While laser therapy remains the primary option among various treatments for acne scars due to its significant efficacy and minimal recovery time, it is important to note that adverse reactions including pain, redness, swelling, and inflammatory pigmentation may manifest post‐treatment.^3^ Our focus lies on exploring an emerging therapy for acne scars known as PDLLA. PDLLA is a synthetic polymer, that is, biocompatible, biodegradable and biostimulatory, promoting dermal collagens production and gradual volume augmentation over time.^14^, ^15^, ^16^, ^17^Studies have indicated that PLLA has the potential to sustain the improvement of rolling acne scars for a duration of up to 4 years.^18^ In addition, treatment with PDLLA has demonstrated a favourable safety profile, with a low risk of nodules or foreign body reactions.^8^ In this study, Juvelook was utilised, comprising 50 mg of particles with sizes below 50 𝜇m, mixed with a small quantity of HA. Juvelook is formulated with larger molecular weights than other PDLLA products and possess a sponge‐like structure, with approximately 80% of the particles filled with air, facilitating moisture retention and decomposition.

PDLLA with small particles is suitable for general dermal regeneration effects and can address issues such as fine wrinkles, skin rejuvenation, whitening, erythema, and photoaging.^13^, ^19^ Conversely, PDLLA with larger particles is typically administered for subcutaneous volume augmentation.^20^, ^21^ However, with this approach, a diluted amount can be injected into the dermis for conditions like depressed scars, yielding superior outcomes compared to manual techniques.

Recent findings from cell and animal experiments have highlighted the superior effects of PDLLA in various aspects of skin regeneration, including extracellular matrix, collagen types I and III regeneration, basement membrane zone regeneration, elastic fibre restoration, stem cell differentiation and mobilisation, and proliferation of papillary dermal fibroblasts, when compared to other collagen stimulators.^15^, ^20^ The division of PDLLA preparations into particles increases the area surrounded by extracellular during immune reactions, thereby benefiting skin regeneration. Moreover, the risk of foreign body reactions is mitigated due to the small volume injected per shot. Our treatment aimed to improve the appearance of atrophic scars, which often necessitate deeper penetration. To achieve this, employing the Mirajet system, allows for the rapid and precise puncturing of the dermis, harnessing a mechanotransduction effect, that is, unachievable through manual injection. This innovative approach is anticipated to gain widespread adoption among dermatologists.

Lofti et al.^22^ conducted a study employing a methodology similar to our approach. This study assessed the efficacy and safety of a combined intervention involving radiofrequency‐assisted subcision and polycaprolactone‐based dermal fillers for treating atrophic postacne scars, targeting various lesion subtypes. The study, which was single‐arm quasi‐experimental in design, included 10 participants with moderate to severe atrophic facial acne scarring. Post‐intervention, significant improvements in both the overall count of acne lesions and specific subtypes were noted. Despite encountering mild adverse events, participants remained engaged in the study. Nevertheless, the study acknowledged limitations, notably a small sample size, emphasizing the need for larger‐scale validation studies. Overall, Lofti et al. suggest that the combined intervention exhibits promise in effectively managing atrophic postacne scars, as evidenced by positive outcomes and participant adherence.

In the study conducted by Ahn et al.^23^ evaluated the efficacy of rotational fractional resection using 1 mm diameter rotating scalpels as a primary treatment for icepick and boxcar scars on the cheeks and glabella region. Three patients underwent a single treatment session of rotational fractional resection, with evaluation 2 months post‐treatment to assess improvements in scar appearance and potential skin‐related side effects. The results revealed significant improvements in the targeted acne scars, with minor suture marks being the only notable skin‐related adverse effects. Despite its limitation in sample size, this study introduces another innovative approach to addressing acne scars, focusing on the removal of fibrous bands and skin tightening, thus avoiding the photothermal harm associated with lasers.

There are several limitations in this study that could be addressed in future research. The small sample size was inadequate to achieve statistical power. Additionally, the study participants were exclusively Korean, with a majority being female. Given the increasing popularity of aesthetic treatments among males, it is important to verify whether the study results are applicable to this demographic as well.^24^ Furthermore, focusing solely on Korean participants with Fitzpatrick skin type III may introduce potential outcome variations when considering populations with different skin phototypes and ethnicities, as previous research has demonstrated variations in skin properties across ethnicities.^25^ Moreover, the lack of standardised volume injected among patients due to challenges in accurately determining the volume of PDLLA injected should be taken into account. Lastly, all patients in this study were treated by the same practitioner, and variations in visible changes may arise due to differences in each practitioner's experience and techniques.

## 5 CONCLUSION

The main visible clinical changes observed in this study with PDLLA delivered by laser‐assisted needle‐free microjet injector improvement of atrophic scars and global improvement of skin quality. Further research, particularly through randomized controlled trials, is needed to validate these findings and assess the longer‐term safety and sustainability of outcomes.

## CONFLICT OF INTEREST STATEMENT

The authors declared no potential conflicts of interest with respect to the research, authorship, and publication of this article. This study was conducted in compliance with the principles set forth in the Declaration of Helsinki.

## Data Availability

The data that support the findings of this study are available from the corresponding author upon reasonable request.
